# Targeting senescence‐associated secretory phenotypes to remodel the tumour microenvironment and modulate tumour outcomes

**DOI:** 10.1002/ctm2.1772

**Published:** 2024-09-13

**Authors:** Jiaqiang Xiong, Lu Dong, Qiongying Lv, Yutong Yin, Jiahui Zhao, Youning Ke, Shixuan Wang, Wei Zhang, Meng Wu

**Affiliations:** ^1^ Department of Obstetrics and Gynecology Zhongnan Hospital of Wuhan University Wuhan China; ^2^ The Second Clinical College of Wuhan University Wuhan China; ^3^ The First Clinical College of Wuhan University Wuhan China; ^4^ Department of Obstetrics and Gynecology Tongji Hospital Tongji Medical College Huazhong University of Science and Technology Wuhan China

**Keywords:** cell senescence, SASP, senolytic, tumour microenvironment

## Abstract

Tumour cell senescence can be induced by various factors, including DNA damage, inflammatory signals, genetic toxins, ionising radiation and nutrient metabolism. The senescence‐associated secretory phenotype (SASP), secreted by senescent tumour cells, possesses the capacity to modulate various immune cells, including macrophages, T cells, natural killer cells and myeloid‐derived suppressor cells, as well as vascular endothelial cells and fibroblasts within the tumour microenvironment (TME), and this modulation can result in either the promotion or suppression of tumorigenesis and progression. Exploring the impact of SASP on the TME could identify potential therapeutic targets, yet limited studies have dissected its functions. In this review, we delve into the causes and mechanisms of tumour cell senescence. We then concentrate on the influence of SASP on the tumour immune microenvironment, angiogenesis, extracellular matrix and the reprogramming of cancer stem cells, along with their associated tumour outcomes. Last, we present a comprehensive overview of the diverse array of senotherapeutics, highlighting their prospective advantages and challenge for the treatment of cancer patients.

**Key points:**

- Senescence‐associated secretory phenotype (SASP) secretion from senescent tumour cells significantly impacts cancer progression and biology.
- SASP is involved in regulating the remodelling of the tumour microenvironment, including immune microenvironment, vascular, extracellular matrix and cancer stem cells.
- Senotherapeutics, such as senolytic, senomorphic, nanotherapy and senolytic vaccines, hold promise for enhancing cancer treatment efficacy.

## Graphical abstract

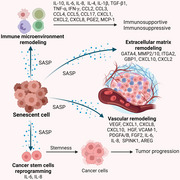

## 1 INTRODUCTION

Cellular senescence, a stress‐induced response, is intricately linked to the aging process and is also implicated in various diseases, including cancer. Some studies have identified senescent cell infiltration in precancerous tissues, particularly in early stage.^1^, ^2^ Additionally, the abnormal activation of oncogenes and exposure to chemotherapy or ionising radiation can induce tumour cell senescence. These senescent cells exhibit some common characteristics, including cell cycle arrest, loss of proliferation biomarkers,^3^ increased senescence‐associated beta‐galactosidase (SA‐β‐gal) activity,^4^ alterations in cell size and morphology,^5^ senescence‐related heterochromatin formation^6^ and sustained DNA damage response (DDR).^7^ Consequently, cellular senescence has traditionally been viewed as a protective barrier against cancer. Additionally, other studies also showed senescent stromal cells can also promote tumorigenesis rather than inhibiting it.^8^, ^9^ Although the tumour‐promoting and anti‐tumour functions of senescence initially appeared incompatible, it was later discovered that most senescent cells secrete a range of inflammatory and growth factors. This secretion, collectively referred to as the senescence‐associated secretory phenotype (SASP), profoundly influences the initiation and progression of tumours.

The SASP comprises a spectrum of soluble factors, including interleukins, chemokines and growth factors, as well as extracellular matrix (ECM) components like integrin, collagen, fibrin, fibronectin and laminin. It also includes proteases such as matrix metalloproteinases (MMPs), urokinase‐type plasminogen activator (uPA) and its receptor (uPAR), non‐macromolecular elements like nitric oxide, reactive oxygen species and extracellular vesicles (EVs) enriched with DNA fragments and miRNAs.^10^, ^11^ Recent evidence suggests that SASP secreted by senescent tumour cells or stromal cells can impact tumour development by remodelling the tumour microenvironment (TME), encompassing vascular changes, immune cell alterations, fibroblast modifications, ECM adjustments and the reprogramming of cancer stem cells (CSCs). Tumour progression and the effectiveness of cancer therapies are influenced by the TME, a complex pathological entity encompassing a diverse array of components.^12^ The SASP serves as a master orchestrator, intertwining the threads that determine the fate of tumours and the outlook for cancer treatments by influencing the TME.

The next step is to unravel the complexity of cellular senescence and SASP in the context of the TME and assess their potential as new therapeutic avenues against cancer. We first introduce the primary triggers of cellular senescence, which include chemotherapy, radiotherapy, oncogene activation, the use of cyclin‐dependent kinase (CDK) inhibitors, telomerase inhibitors and agents that target epigenetic regulation. Subsequently, we delve into the regulatory mechanisms of SASP secretion and focus on the impact of SASP on TME (macrophage, T‐cell, natural killer [NK] cells, myeloid‐derived suppressor cells (MDSCs), vascular remodelling, ECM remodelling and CSCs reprogramming) and tumour prognosis. Finally, we offer a comprehensive summary of senotherapy for tumour senescence and discuss future prospects and possible challenges for senescence‐associated treatment.

## 2 THE INDUCEMENT OF CELLULAR SENESCENCE IN TUMOUR

**FIGURE 1:**
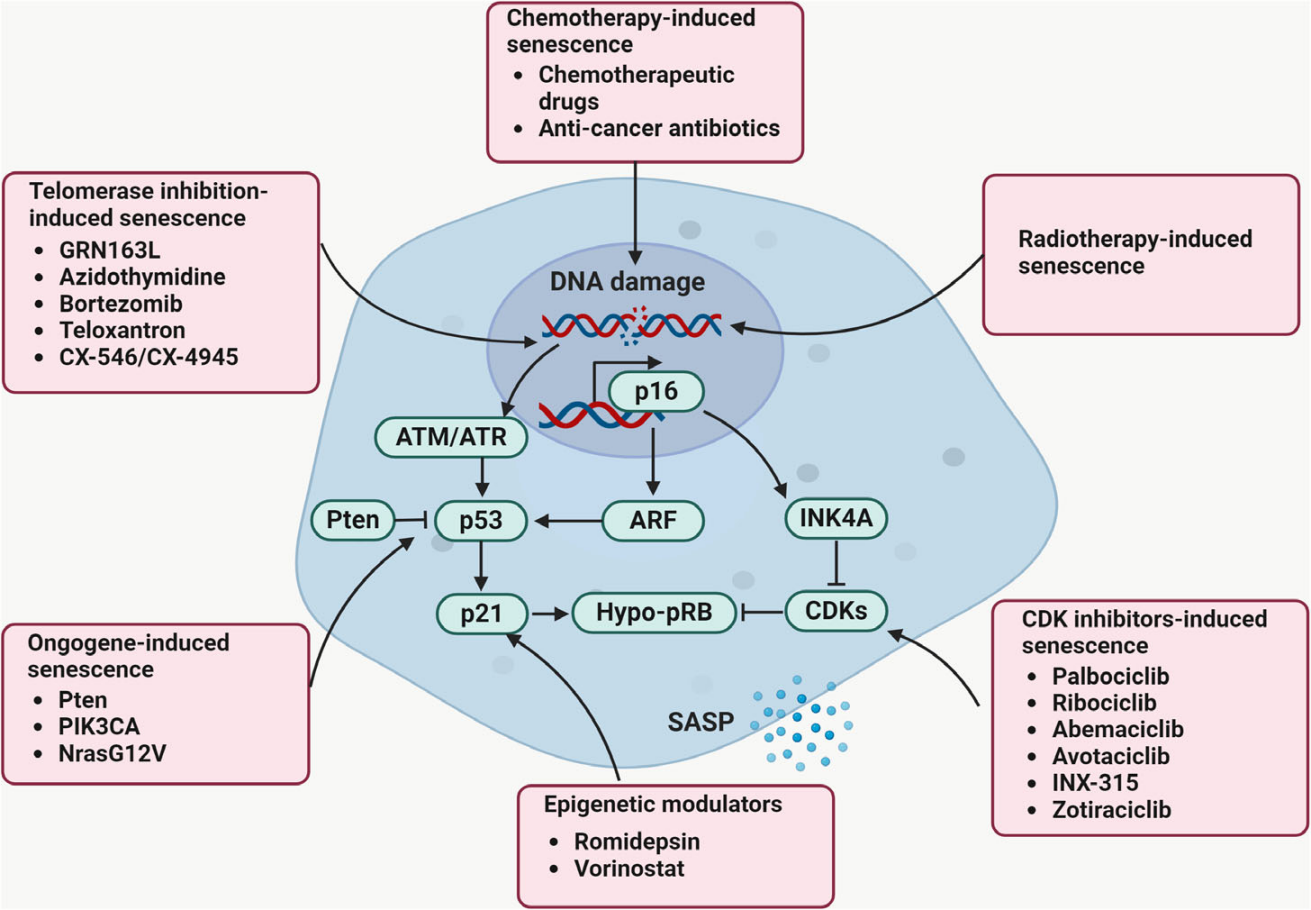
The inducement of cellular senescence in tumours. Treatments such as chemotherapy, radiotherapy, cyclin‐dependent kinase (CDK) inhibitors, telomerase inhibitors and epigenetic modulators have the potential to induce cellular senescence in both cancer cells and stromal cells. Persistent DNA damage caused by chemotherapies, radiotherapy and telomerase inhibitor treatment often results in induction of senescence. Massive DNA damage response triggers ATM or ATR signalling and results in p53 and p21 activation. Consequently, cells undergo senescence via hypophosphorylation of RB (hypo‐pRB). p53–p21 signalling‐mediated senescence can also be triggered by oncogenic signals. CDK inhibition‐mediated senescence can be induced by inhibitors of CDK1, CDK2, CDK4/6, CDK7, CDK9 and CDK8/19.

Cellular senescence exerts profound effects not only on tumour development but also on responses to anti‐cancer therapies. The principal mediators of proliferation arrest associated with senescence are the CDK inhibitors p21 and p16INK4a, which impede the assembly of CDK–cyclin complexes crucial for cell cycle progression at the G1–S phase transition. Notably, in addition to certain oncogenes capable of inducing tumour cell senescence, specific anti‐tumour therapies, including chemotherapy, radiotherapy, CDK inhibitors, telomerase inhibitors and agents that target epigenetic regulation (Figure 1), have been demonstrated to increase the presence of senescent cells or the expression of senescence markers.

### 2.1 Oncogene‐induced senescence

The initial confirmation of oncogene‐induced senescence (OIS) occurred in 1997 when the ectopic expression of the Ras allele (H‐Ras V12) in human fibroblasts (IMR90) led to growth arrest.^13^ Later, the concept of OIS was extended to diverse carcinogenic models. For instance, the H1047R mutation in the oncogenic PIK3CA gene in breast cancer enhances PI3K enzyme activity, activating the AKT/mTOR signalling pathway and inducing the glycosylation of membrane metalloendopeptidase, ultimately resulting in tumour cell senescence.^14^

The effects of OIS on tumour cells are linked to the balance between immune monitoring and immune escape. In mouse models of senescence driven by NrasG12V, senescence not only inhibits the proliferation of tumour cells, but also activates CD4^+^ T cells to eliminate tumour cells.^15^ Additionally, in HrasG12V‐mutated hepatocellular carcinoma (HCC), senescent HCC cells secrete SASP factors (such as CCL2, IL‐15 and CXCL1) that activate macrophages, neutrophils and NK cells, thereby mediating innate immunity and suppressing tumour progression.^16^ Nevertheless, immune regulation in the later stages can foster tumour invasion, resulting in the up‐regulation of inhibitory checkpoints and the recruitment of immunosuppressive cells. For instance, CCL2, secreted by precancerous senescent hepatocytes, which are inadequately cleared by the immune system subsequent to NrasG12V‐induced senescence, can recruit immature myeloid cells (CD11b^+^, Gr‐1^+^) to impair NK cell function and promote oncogenesis.^17^ Conversely, in another study, Jak2/Stat3 inactivation within Pten‐null senescent tumours alters the immunosuppressive properties of SASP by decreasing the levels of specific cytokines (such as CXCL2, GM‐CSF, M‐CSF, C5a, IL‐10, IL‐13) in the TME, thereby facilitating the transition from immunosuppression to active immune surveillance.^18^ In summary, the impact of OIS‐induced senescence on tumours is dynamic and intricately linked to the SASP and the accompanying immune status, and a persistent state of cellular senescence may be a negative factor.

### 2.2 Chemotherapy‐induced senescence

Modulating the dosage of chemotherapy drugs can induce tumour cell senescence and inhibit tumour development. Low‐dose chemotherapy induces cell senescence via DNA damage and an extended DDR mediated by ATM/ATR, whereas high‐dose chemotherapy triggers cell apoptosis.^19^ This phenomenon elucidates why only certain tumour cells undergo senescence following conventional chemotherapy. Mechanistically, numerous chemotherapy drugs induce tumour cell senescence through the ATR–CHK1 and ATM–CHK2 pathways, activating the senescence‐associated pathways involving p53–p21 and p16–RB (Figure 1).^20^, ^21^ Alkylating agents like cyclolinamide, ifosfamide and busulfan, for instance, create DNA cross‐links by covalently binding to electronic groups in DNA, thereby inducing senescence through DDR.^22^ Likewise, non‐cell cycle‐specific drugs, including carboplatin, cisplatin and oxaliplatin, induce DNA damage and cell senescence through DNA cross‐linking.^23^

While chemotherapy drugs can induce tumour cell senescence, the SASP secreted by these cells exerts varying effects on tumour progression within the TME. Cyclophosphamide treatment activates NK cell‐mediated immune surveillance in B cell lymphoma models through an NF‐κB‐mediated SASP.^24^ In another study, the SASP secretion of IL‐1β, IL‐8 and CXCL10 induced by cisplatin and irinotecan renders ovarian cancer responsive to programmed cell death 1 (PD‐1) treatment, triggering the activation of CD8^+^ T cells and enhancing dendritic cell infiltration.^25^ In multiple myeloma, low‐dose doxorubicin and melphalan induce the expression of NK cell‐activating ligands on senescent tumour cells, thereby enhancing NK cell recognition and cytotoxicity against the tumour cells.^26^, ^27^ This illustrates that senescent tumour cells post‐chemotherapy could serve as immune adjuvants, potentiating anti‐tumour immune responses. Conversely, chemotherapy‐induced senescence can also induce immune suppression within tumours. In a prostate cancer model, treatment with docetaxel led to the suppression of NK and T cells, promoting chemoresistance through the induction of an anti‐inflammatory SASP.^18^ In total, at the beginning of treatment, most tumours are sensitive and effective to chemotherapy; however, as the number of senescent tumour cells increases, chemotherapy‐induced senescent tumour cell may transcriptional reprogram as a mechanism of stress adaptation that renders cells less drug‐responsive and prone to generating aggressive progeny, finally resulting in a poor prognosis.

Chemotherapy‐induced senescent tumour cells not only secrete SASP but also exhibit altered expression of immune checkpoint molecules. Recently, an unbiased proteomics analysis has identified a significant up‐regulation of the immune checkpoint inhibitor, programmed cell death 1 ligand 2 (PD‐L2), in various chemotherapy‐induced senescent cancer cells.^28^ The study also demonstrated that the therapeutic blockade of PD‐L2 with antibodies potently enhances the efficacy of chemotherapy, leading to the remission of mammary tumours in a murine model. The combination of chemotherapy with anti‐PD‐L2 treatment offers a therapeutic strategy that capitalises on the vulnerabilities that emerge from therapy‐induced cellular senescence.

Chemotherapy could result in the accumulation of senescent cells within both malignant and non‐malignant tissues. In clinical studies, the number of T cells overexpressing p16INK4A was increased in patients treated with chemotherapeutic agents, which suggests that immune‐senescence may arise incidentally as a side effect of the treatment process.^29^, ^30^, ^31^, ^32^ One explanation posits that chemotherapy, acting as a DNA‐damaging agent, induces T cell senescence through the up‐regulation of γH2AX and p16, canonical biomarkers indicative of senescence in somatic cells.^33^ Notably, senescent T cells exhibit diminished cytotoxic capabilities and may contribute to an immunosuppressive TME, which further lead to a poor response.

### 2.3 Radiotherapy‐induced senescence

Radiotherapy is predominantly employed for localised treatment in various cancer types. Radiotherapy can induce senescence by causing double‐stranded DNA breaks (DSBs), subsequently activating the ATM/ATR–CHEK2–p53 pathway and its downstream target, p21 (Figure 1).^19^, ^34^ Both in vitro and in vivo experiments, along with preclinical evidence, suggest that senescence significantly contributes to radiosensitivity in cancer.^35^, ^36^, ^37^

MDA‐MB231 breast cancer cells with mutant p53 fail to undergo senescence following radiotherapy, ultimately resulting in apoptosis. This indicates the essential role of p53 in radiation‐induced senescence.^35^ Furthermore, radiotherapy has been demonstrated to induce retinoblastoma (RB)‐dependent senescence and the secretion of IL‐6, consequently enhancing immune surveillance by NKT cells in osteosarcoma.^37^ DNA damage induced by radiotherapy and the accumulation of cytoplasmic DSBs also activate the cGAS–STING pathway and its downstream NF‐κB pathway, leading to the production of SASP and enhanced anti‐tumour effect.^38^ Additionally, irradiation can stimulate lung cancer cells to release SASP through CD63‐positive EVs containing DNA:RNA hybrids and LINE‐1 retrotransposon, thereby inducing senescence in distant non‐irradiated cells and enhancing the efficacy of radiotherapy.^39^ However, irradiation increases the burden of senescent cells in the normal tissue surrounding the cancer site. This can lead to local side effects, including immunosuppression and stromal senescence‐associated tumorigenesis.^40^ In an immune‐deficient glioblastoma model, radiation‐induced senescence accelerated tumour growth, indicating that the elevated presence of senescent cells and SASP following irradiation is a primary factor contributing to tumour recurrence in glioblastoma patients.^36^ These findings underscore the complex and multifaceted role of senescence and SASP in response to radiotherapy, emphasising the need for a comprehensive understanding of these processes in both preclinical and clinical settings.

Similar to chemotherapy, radiation has been demonstrated to trigger senescence in non‐malignant cells. Microarray hybridisation analysis revealed an elevated expression of p21 in the leukocytes of patients who underwent radiotherapy.^41^ In a clinical study, the presence of a high number of circulating CD8^+^CD28^−^ senescent T cells have been shown to be an independent predictor of distant metastasis in patients with nasopharyngeal carcinoma after radiotherapy.^42^ This correlation may be associated with the tumour's ability to evade immune detection and response. However, it is worth clarifying whether the radiotherapy is engaged in regulating T cell senescence.

### 2.4 CDK inhibitors‐induced senescence

CDK governs cell cycle checkpoints, dictating the growth or arrest of both tumour and non‐tumour cells.^19^ A defining feature of senescent cells is the elevation of CDK suppressor proteins like p16 and p21. These proteins activate RB and impede cell cycle progression, culminating in cellular senescence.^9^ The proliferation and metastasis of cancer cells require overcoming the hindrances posed by cellular senescence. This is achieved by increasing the expression levels of CDK.^19^, ^43^ Hence, investigations into drugs that either inhibit CDK or enhance the levels of CDK suppressor proteins are underway for cancer therapy. The current roster of common CDK inhibitors encompasses a range of subtypes, including those targeting CDK1, CDK2, CDK4/6, CDK7, CDK8/19 and CDK9. Notably, CDK4/6 inhibitors have obtained regulatory approval from the United States Food and Drug Administration (US FDA), while inhibitors of other CDK subtypes are actively undergoing evaluation in clinical trials.

CDK4/6 inhibitors exert their effects by disrupting the formation of complexes between CDK4/6 and cyclin D, thereby impeding ATP binding. This intervention effectively attenuates upstream growth signals, halting the cell cycle's progression from the G1 phase to the S phase. As a result, CDK4/6 inhibition promotes cell senescence and apoptosis, offering a therapeutic strategy for certain cancers. The most extensively studied CDK inhibitors proved by US FDA in clinical settings are the dual CDK4/6 inhibitors, comprising palbociclib, ribociclib, abemaciclib and trilaciclib. Treatment with CDK4/6 inhibitors can induce senescence in specific cancer cells, including oestrogen receptor (ER)‐positive breast cancer, lung cancer, melanomas and liposarcoma.^19^, ^44^, ^45^, ^46^, ^47^, ^48^ In breast cancer models, treatment with abemaciclib induced SA‐β‐gal activity and resulted in heightened infiltration of cytotoxic CD8^+^ T cells.^49^ Likewise, abemaciclib treatment reversed intrinsic immune suppression within tumours in human melanoma patients. It induced components of the SASP, including CCL20 and CXCL1, consequently enhancing the efficacy of combined anti‐PD‐1/CTLA‐4 in melanoma models.^45^ The combination of CDK4/6 inhibitors with other targeted therapies additionally promotes tumour immunity by enhancing cellular senescence in tumour cells. Palbociclib, when combined with a mitogen‐activated protein kinase (MAPK) inhibitor, can effectively induce senescence and elicit a robust SASP in KRAS‐driven lung cancers, initiating a surveillance program by NK cells that ultimately results in tumour cell death.^48^ The combined MEK and CDK4/6 inhibition induced tumour senescence and SASP‐driven endothelial cell activation, fostering the accumulation of CD8^+^ T cells and rendering tumours more responsive to PD‐1 checkpoint blockade.^50^ These findings collectively underscore the multifaceted role of CDK4/6 inhibitors in shaping the TME through inducing senescence and SASP secretion.

Avotaciclib (BEY1107), an orally bioavailable CDK1 inhibitor, is currently under investigation in Phase I/II clinical trials. It is being assessed both as a standalone treatment and in conjunction with gemcitabine for patients with locally advanced or metastatic pancreatic cancer (NCT03579836). Some studies have shown that inhibition of CDK1 can promote cell senescence by inducing cell cycle arrest.^51^, ^52^ However, the activation of CDK1 is also a necessary step in the regulation of cell senescence, especially during the process of mitosis.^53^, ^54^ Overall, the role of CDK1 in cell senescence may be dualistic, capable of both promoting and preventing cell senescence under certain conditions, depending on the specific cellular environment and the contextual regulation of CDK1 activity.

INX‐315, as a targeted CDK2 inhibitor, has demonstrated the ability to induce cell senescence in solid tumours. This compound provides durable control over tumour growth and has shown promise in overcoming resistance to CDK4/6 inhibitors in breast cancer.^55^ Furthermore, zotiraciclib, an efficacious oral CDK9 inhibitor with the capacity to penetrate the blood–brain barrier, has demonstrated the ability to degrade the MCL‐1 and Myc oncoproteins. This action promotes apoptosis and senescence, particularly within the context of glioma treatment.^56^, ^57^ Additionally, other CDK inhibitors, such as those targeting CDK7^58^ and CDK8/19,^59^ have also shown promise in inducing tumour cell senescence, offering a broadening scope of therapeutic options in oncology.

### 2.5 Telomerase inhibition‐induced senescence

Telomeres, serve as crucial protective structures that shield chromosome ends from degradation and illegitimate recombination, playing a pivotal role in cellular fate and the aging process. Telomerase, a specialised reverse transcriptase, employs an intrinsic RNA template to extend the telomeric G‐rich strand. Remarkably, over 85% of human cancers exhibit telomerase activity, characterised by increased telomerase activity and heightened expression of human telomerase reverse transcriptase (hTERT).^60^ Therefore, targeting telomerase has emerged as a promising strategy for anti‐cancer interventions.

A multitude of therapeutic strategies have been devised to target cancer cells that rely on telomerase activity. These approaches encompass hTERT‐targeting DC vaccines, virotherapy utilising oncolytic viruses specific to telomerase, telomerase‐targeted peptide vaccines, hTERT‐targeting gene therapy involving recombinant viral or plasmid vectors and telomerase‐mediated CRISPR/Cas9 technology.^61^, ^62^, ^63^ Furthermore, various drugs have been developed to inhibit telomerase activity and induce senescence in tumour cells. Imetelstat, the sole US FDA‐approved telomerase inhibitor, is utilised in the treatment of myelofibrosis. Notably, in human pancreatic cancer cells, treatment with the telomerase inhibitor imetelstat (GRN163L) resulted in lifespan limitation, accompanied by the activation of a DDR marked by γ‐H2AX and an increase in senescence, as evidenced by SA‐β‐gal activity.^64^ Furthermore, bortezomib (BTZ), a proteasome inhibitor, reduced the expression levels of hTERT and induced cell cycle arrest with a significant increase in β‐Gal activity in A549 lung cancer cells.^65^ Teloxantron, a potent small‐molecule telomerase inhibitor from anthraquinone derivative, activates DSBs by the inhibition of telomerase, which triggers replicative senescence and cell apoptosis in TERT‐positive lung cancer cell involving ATM/Chk2 and ATR/Chk1 pathways.^66^ Azidothymidine, initially used to treat AIDS, represents one of the earliest reported telomerase inhibitors, with its application extending to phase II clinical trials for anti‐cancer therapy.^67^ Similarly, CX‐4945 and CX‐5461, recognised for their ability to block telomerase activity, have undergone phase I/II clinical trials and garnered significant acclaim within the field of cancer research.^68^ Nevertheless, the utilisation of these telomerase inhibitors to promote pro‐senescence effects in tumours warrants further scrutiny and investigation.

### 2.6 Epigenetically induced senescence

Epigenetic modifications, including alterations to DNA and histones, serve as potent regulators of gene expression. These modifications are integral to the processes that induce and sustain cellular senescence.

DNA methylation, a potent epigenetic modification, facilitates transcriptional silencing. In replicative senescence, a global hypomethylation was observed in senescent cells compared with proliferating cells, although certain genes paradoxically undergo hypermethylation during cellular senescence.^69^ Senescent cells also undergo significant alterations in chromatin architecture, notably the formation of senescence‐associated heterochromatin foci, indicating that hypomethylation and concomitant chromatin reconfigurations contribute to the senescence phenotype.^70^ Moreover, the induction of cell senescence by sulforaphane is correlated with DNA hypomethylation, with reduced levels of DNA methyltransferases (DNMT1, DNMT3B) in breast cancer cells.^71^

Histone methylation can activate or inhibit gene expression and relate to cell senescence. Recently, researchers have demonstrated that down‐regulation of enhancer of zeste homolog 2 (EZH2), functioning as an H3K27 methyltransferase, can trigger senescence and the SASP through DNA replication‐dependent DNA damage, concurrent with induction of p21.^72^ Furthermore, the PLK4–H3K27me3 axis has been linked to histone methylation, and sustained inhibition of PLK4 has been shown to impede the growth of TP53‐mutated acute myeloid leukaemia, leading to DNA damage, apoptosis and senescence.^73^

There is a complex interplay between histone acetylation and cellular senescence, which may serve as a potential target for anti‐aging therapy. Histone deacetylases (HDACs) are enzymes that predominantly function to deacetylate histones, thereby regulating gene expression. In humans, 18 distinct HDACs have been identified and are categorised into four classes across two families. Several HDAC inhibitors, including panobinostat, romidepsin, vorinostat, belinostat and tucidinostat, have received clinical approval and are utilised in the treatment of T‐cell lymphoma, breast cancer and multiple myeloma.^74^ The application of some HDAC inhibitors can lead to senescence in tumour cells. For instance, romidepsin induces cell senescence in pancreatic cancer through the down‐regulation of FOXM1 expression.^75^ Additionally, vorinostat has been demonstrated to induce senescence in human dermal fibroblasts, as evidenced by the up‐regulation of P16 and P21, as well as an increase in SA‐β‐gal activity.^76^ Furthermore, the combination of vorinostat and cisplatin has proven exceptionally effective in eliminating CSCs and reducing tumour viability in adenoid cystic carcinoma by triggering cellular senescence.^77^ Conversely, belinostat has been observed to counteract irradiation‐triggered senescence in HaCaT cells by suppressing the activation of the UVB‐induced NF‐κB/p65 signalling cascade.^78^ The exploration of HDAC inhibitors and their impact on cellular senescence remains a topic of significant interest and potential therapeutic promise.

## 3 REGULATION OF SASP INDUCTION IN TUMOUR

Various factors can regulate the secretion of SASP by senescent cells, including cell type, duration of stimulation, stimulating factors and the extent of injury.^79^ Multiple mechanisms, including NF‐κB signalling, p38MAPK signalling, mTOR signalling, ATM signalling and autophagic activity, have been reported to mediate these regulatory effects in senescent tumour cells.

Components of the DDR pathway and DNA repair proteins play essential roles in initiating and sustaining SASP. For example, in RAS‐induced senescence, ATM is not essential for cell growth arrest, but its absence abolishes the secretion of the SASP factor IL‐6.^80^ Recent research has identified the transcription factor GATA binding protein 4 (GATA4) as an upstream activator of NF‐κB, initiating SASP through the DDR kinases ATM and ATR.^81^ Prolonged ATM activation triggers the degradation of histone methyltransferases, resulting in epigenetic derepression of a subset of SASP genes and subsequent SASP secretion.^82^ In essence, persistent DDR appears to provoke SASP through ATM or ATR signalling.

Senescent cells also undergo epigenetic modifications, which are critical for modulating the expression of SASP genes. For example, histone variants such as macroH2A1 and H2AJ are up‐regulated in OIS fibroblasts, positively modulating the expression of a broad spectrum of SASP genes.^83^, ^84^ In addition, HDAC inhibitors stimulate the expression of a multitude of SASP factors in senescent fibroblasts.^85^ Studies have revealed that both SIRT1 and EZH2 negatively regulate SASP secretion in senescent cells through modifications of histones.^72^, ^86^ High mobility group box 2, a non‐histone chromatin‐bound protein, selectively accumulates at and up‐regulates SASP loci, such as IL‐6 and IL‐8.^87^ Other epigenetic regulators, such as mixed‐lineage leukaemia 1 (MLL1) and bromodomain‐containing protein 4 (BRD4), also play crucial roles in the regulation of SASP.^88^, ^89^ MLL1 is critical for the expression of genes that promote proliferation, which is necessary to trigger the DDR and subsequently induce SASP.^88^ In contrast, BRD4 binds to super‐enhancer regions enriched in proximity to key SASP genes, accelerating SASP induction.^89^ These findings collectively suggest that SASP induction is subject to regulation through epigenetic changes occurring in senescent cells.

## 4 SASP AND TUMOUR MICROENVIRONMENT REMODELLING

**FIGURE 2:**
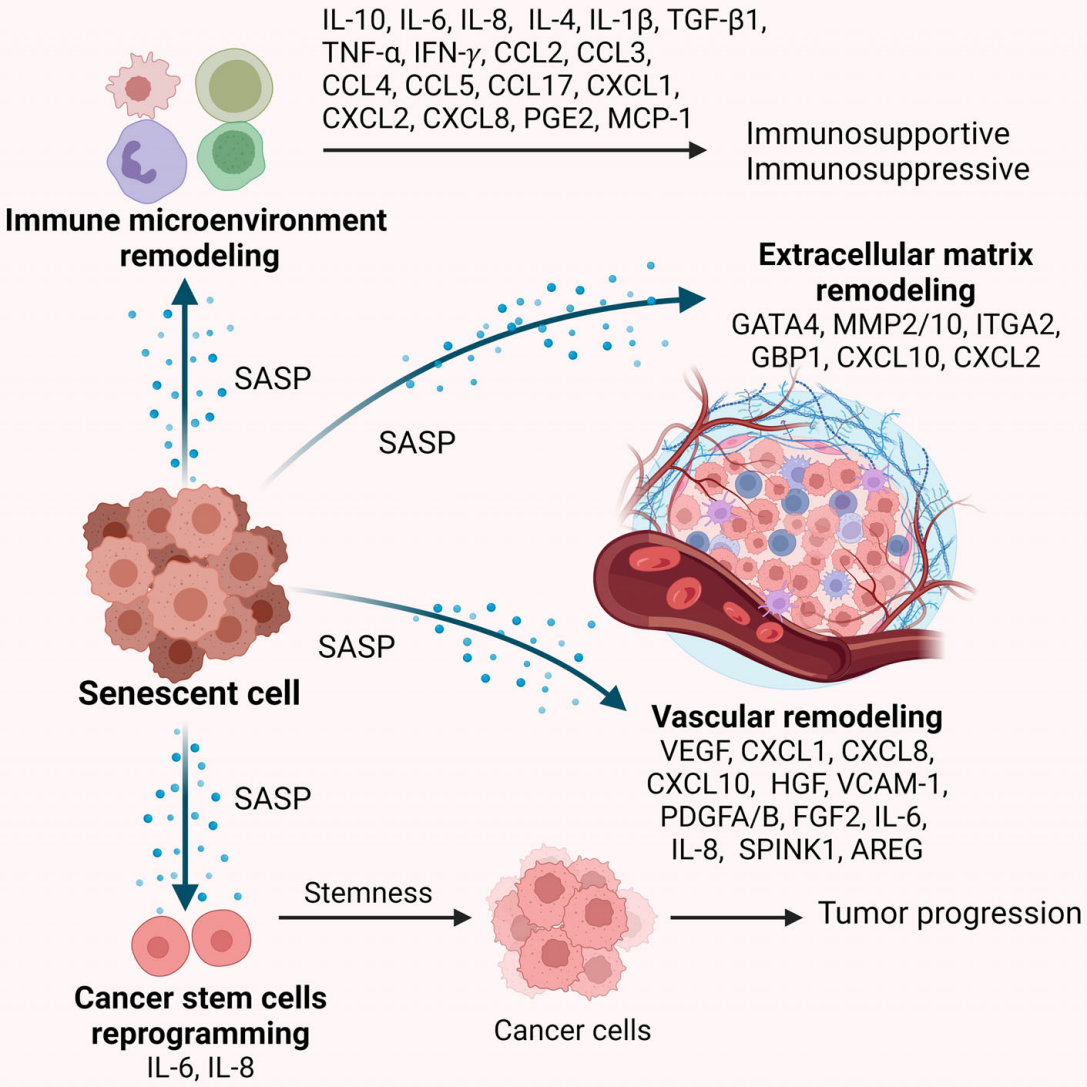
Roles of SASP in tumour microenvironment remodelling. SASP such as IL‐10, IL‐6, IL‐8, IL‐4, IL‐1β, TGF‐β1, TNF‐α, IFN‐γ, CCL2, CCL3, CCL4, CCL5, CCL17, CXCL1, CXCL2, CXCL8, PGE2 and MCP‐1 contribute to immune microenvironment remodelling. Meanwhile, GATA4, MMP2/10, ITGA2, GBP1, CXCL10 and CXCL2 are involved in extracellular matrix remodelling. Factors like VEGF, CXCL1, CXCL8, CXCL10, HGF, VCAM‐1, PDGFA/B, FGF2, IL‐6, IL‐8, SPINK1 and AREG contribute to vascular remodelling. Additionally, IL‐6 and IL‐8 are implicated in CSCs reprogramming. Ultimately, these SASP‐mediated alterations in the tumour microenvironment can either facilitate or suppress tumorigenesis.

Once the senescence process is activated and the arrest of cell cycle is irreversible, early cancer development must overcome the obstacles imposed by cell senescence.^79^ Moreover, senescent cells are capable of recruiting immune cells and inducing senescence in adjacent cells, thereby inhibiting or promoting tumour initiation.^19^, ^90^ Here, we discuss how senescent cells, influence the TME through the SASP, including its impact on the immune system, ECM, the vascular compartment and CSCs (Figure 2). The bidirectional effects of the SASP secreted by senescent cells in TME remodelling partly elucidate the tumour‐promoting or tumour‐suppressing effects during tumour development.

### 4.1 SASP‐induced immune microenvironment remodelling

Senescence triggers the remodelling of immune system within TME via the modulation of a spectrum of cytokines, chemokines, growth factors and proteinases, collectively termed the SASP. The impact of SASP on immune system varies depending on the cell type and the process of cellular senescence induction. While SASP exhibits immune‐activating properties in specific TME, it is noteworthy that SASP factors can also, under certain circumstances, suppress host immunity.

#### 4.1.1 SASP and macrophage

SASP, secreted by senescent tumour cells through various signalling pathways, can recruit macrophages, leading to their infiltration into the TME. Inhibition of the PI3K/AKT/mTOR axis has been shown to elicit senescence in breast cancer, resulting in the secretion of SASP factors (CCL2, CXCL1, CXCL8, IL‐6), which induce an M2‐like polarisation.^14^ Similarly, Chung et al.^91^ demonstrated that ionising radiation‐induced senescent lung cancer cells can polarise macrophages towards the M2 phenotype, and this polarisation is dependent on IL‐13, regulated by the IGF‐1 signalling pathway. Additionally, exposure of human monocytes to conditioned medium from thyroid cancer cell lines leads to the polarisation of M2 macrophages, induced by PGE2 and CCL17.^92^ Senescent thyroid cancer cells induced by H2O2 activate the IKK2/IkB/p65 signalling pathway, resulting in the secretion of CXCL2, CXCL3 and IL8, which further drives the polarisation of M2 macrophages.^93^ In chemotherapy‐induced senescent colon tumour cells, the elevated M‐CSF expression promotes the polarisation of macrophages from the M1 to M2 phenotype.^94^ In Pten‐null senescent tumours, the JAK2/STAT3 pathway was activated, resulting in the secretion of CXCL1, CXCL2 and IL‐6, thereby promoting macrophage M2 polarisation.^18^ While the specific chemokines and interleukins secreted by senescent tumour cells may vary in these diverse cancer cell types, they all play a pivotal role in macrophage polarisation towards the M2 phenotype.

Although the majority of studies mentioned above have focused on the polarisation of macrophages into the M2 phenotype, some research reveals the correlation between SASP and the M1 phenotype. SASP secreted by senescent tumour cells primarily influences macrophage autophagy and the phosphorylation of Stat1/Stat6, leading to a shift in macrophage phenotype towards the M1 state. For example, SASP components (IL‐1β, IL‐6, TNF‐α and CCL2) can regulate macrophage autophagy by recruiting neutrophils, inducing macrophage M1 polarisation.^95^ Furthermore, in hepatic stellate cells, p53‐induced senescence triggers the production of SASP factors including IL‐6, ICAM1 and IFN‐𝛾, which promote macrophage polarisation shifting from an immune‐suppressive M2 state to an immune‐stimulatory M1 state.^96^ Similarly, in non‐small cell lung cells undergoing p53‐induced senescence, the release of SASP factors such as IL‐6 and IL‐1α contributes to macrophage polarisation towards the M1 phenotype.^39^

Collectively, these findings indicate that the SASP can orchestrate varied immune responses, including both anti‐tumour effects (M1 macrophages) and pro‐tumour activities (M2 macrophages) (Table 1). This process may involve epigenetic changes in senescent tumour cells and macrophages, influenced by multiple factors such as cell type, stimulus factors, stimulation duration and the extent of damage. Further exploration is needed to elucidate how SASP regulates the function and polarisation of macrophages.

**TABLE 1:** The role of SASP in tumour microenvironment.

Senescence trigger	Type of cancer	Pathway	SASP factors	Effect	Polarisation of macrophage	References
OIS (PIK3CA)	Breast	AKT/mTOR	CCL2, CXCL1, CXCL8, IL‐6	Pro‐tumour	M2 polarisation	^14^
Irradiation	Lung	IGF‐1	IL‐13	Pro‐tumour	M2 polarisation	^91^
Doxorubicin	Colon	iASPP‐Nrf2	MMP3, MCP‐1, M‐CSF, IL‐10	Pro‐tumour	M2 polarisation	^94^
OIS (Pten)	Prostate	JAK2/STAT3	CXCL1, CXCL2, IL‐6	Pro‐tumour	M2 polarisation	^18^
Tamoxifen	Thyroid	COX‐2	PGE2, CCL17	Pro‐tumour	M2 polarisation	^97^
H2O2	Thyroid	NF‐κB	CXCL2, CXCL3, IL‐8	Pro‐tumour	M2 polarisation	^93^
OIS (RAS)	Thyroid	COX‐2	PGE2, CSF‐1	Pro‐tumour	M2 polarisation	^92^
Doxorubicin	Liver	TP53	IL‐6, ICAM1, IFN‐γ	Anti‐tumour	M1 polarisation	^96^
Irradiation	Non‐small cell lung	TP53	IL‐6, IL‐1α	Anti‐tumour	M1 polarisation	^39^
Trametinib, palbociclib	Pancreas	ND	VEGF, PDGFA/B, FGF2, MMPs (MMP2/3/7/9/10)	Anti‐tumour	Increased CD8^+^ T cells	^50^
OIS (Nras)	Liver	MAPK	CTACK, IL‐1α, leptin, MCP1, RANTES	Anti‐tumour	Increased CD4^+^ T cells	^15^
CDK4/6 inhibitors	Melanoma	ND	IFN‐γ, IFN‐β	Anti‐tumour	Increased CD8^+^ T cells	^45^
Cisplatin, irinotecan	Ovary	cGAS	IL‐1β, IL‐8, CXCL10	Anti‐tumour	Increased CD8^+^ T cells and DCs	^25^
Doxorubicin	Melanoma	MHC‐I	Senescence‐associated MHC‐I‐peptides	Anti‐tumour	Increased CD8^+^ T cells and DCs	^98^
OIS (Pten)	Prostate	JAK2/STAT3	CXCL1, CXCL2, IL‐6	Pro‐tumour	Increased MDSCs, reduced activity of T cells	^18^
CDK4/6 inhibitors	Breast	E2F	IL‐29, IL‐28a, IL‐28b	Anti‐tumour	Inhibit the proliferation of Treg cells	^99^
OIS (RB1), irradiation	Osteosarcoma	ND	IL‐6	Pro‐tumour	Decreased NKT cells	^37^
OIS (p53)	Liver	p53	CSF1, MCP1, CXCL1, IL‐15	Anti‐tumour	Increased NK cells	^100^
OIS (p53)	Liver	p53	CCL2, CCL3, CCL4, CCL5	Anti‐tumour	Increased NK cells	^16^
MAPK and CDK 4/6 inhibitors	Lung	RB	CCL2, CCL4, CCL5, CXCL10, CXCL1	Anti‐tumour	Increased NK cells	^48^
OIS (Nras)	Liver	CCL2–CCR2	CCL2	Pro‐tumour	Increased MDSCs, decreased NK cells	^17^
OIS (p27)	Squamous cell carcinoma	ND	IL‐6	Pro‐tumour	Increased MDSCs	^40^
CDK4/6 inhibitor	Melanoma	NF‐κB	IL‐6, MMP3, CCL6, CCL8, CCL11	Pro‐tumour	Increased MDSCs	^101^

#### 4.1.2 SASP and T cells

T cell infiltration within the TME correlates with the survival rates of cancer patients. Senescent tumour cells secrete SASP, which not only recruits T cells but also regulates their activity, influencing immune surveillance and tumour development.

One crucial mechanism for tumour inhibition involves increasing the sensitivity of tumour cells to immune checkpoint blockade (ICB). In therapy‐induced tumour senescence, there is an enhanced expression of pro‐angiogenic factors (FGF2, VEGF and PDGFA/B) and MMPs (MMP2/3/7/9/10). These factors stimulate the accumulation of CD8^+^ T cells within tumours that are otherwise considered immunologically ‘cold’, making pancreas tumours more responsive to ICB.^50^ Mouse models of NrasG12V‐driven senescence demonstrate that senescent hepatocytes secrete chemokines and cytokines (CTACK, IL‐1α, leptin, CCL2 and RANTES), promoting a CD4^+^ T‐cell‐mediated adaptive immune response.^15^ In senescent melanoma, CDK4/6 inhibitors increase the secretion of IFN‐γ and IFN‐β, attracting infiltrating CD8^+^ T cells into the TME and sensitising tumour cells to immune checkpoint inhibitors.^45^ Additionally, the combination of cisplatin and irinotecan enhances the secretion of IL‐1β, IL‐8 and CXCL10 through the cGAS pathway, which promotes the infiltration of CD8^+^ T cells and dendritic cells, effectively inhibiting tumour growth.^25^ In a recent study, senescent cancer cells can be harnessed to foster potent and protective CD8^+^ T cells‐mediated anti‐tumour immune responses.^98^ The influence of SASP on T cells extends beyond early recruitment, also affecting T cell function. Senescent prostate cancer cells secrete IL‐6, CXCL1 and CXCL2, which inhibit the activity of T cells, including both CD4^+^ T and CD8^+^ T cells, thereby promoting prostate tumour growth.^18^

Regulatory T cells (Treg cells) play a crucial role in tumour proliferation and metastasis by producing various immunosuppressive cytokines that hinder the immune response.^12^ In canine oral melanocytes, senescent cancer cells induce the differentiation of Tregs and promote tumour growth through TGF‐β1.^102^ At the same time, studies have found that CDK4/6 inhibitors inhibit the proliferation of Treg cells by inducing the secretion of IL‐29, IL‐28a and IL‐28b from senescent breast cancer cells, overcoming tumour immune evasion and enhancing anti‐tumour immunity.^99^

NKT cells, a distinct subgroup of T cells, play important roles in anti‐tumour immunity.^103^ IL‐6 secreted by RB1‐dependent senescent osteoblasts following ionising radiation actives NKT cell enrichment along with a host inflammatory response, and mice devoid of IL‐6 or NKT cells demonstrate a rapid progression in the development of IR‐induced osteosarcomas.^37^ In a mouse model of HCC, CXCR6‐mediated NKT cell infiltration eliminates senescent hepatocytes, thereby inhibiting tumour development.^104^

**FIGURE 3:**
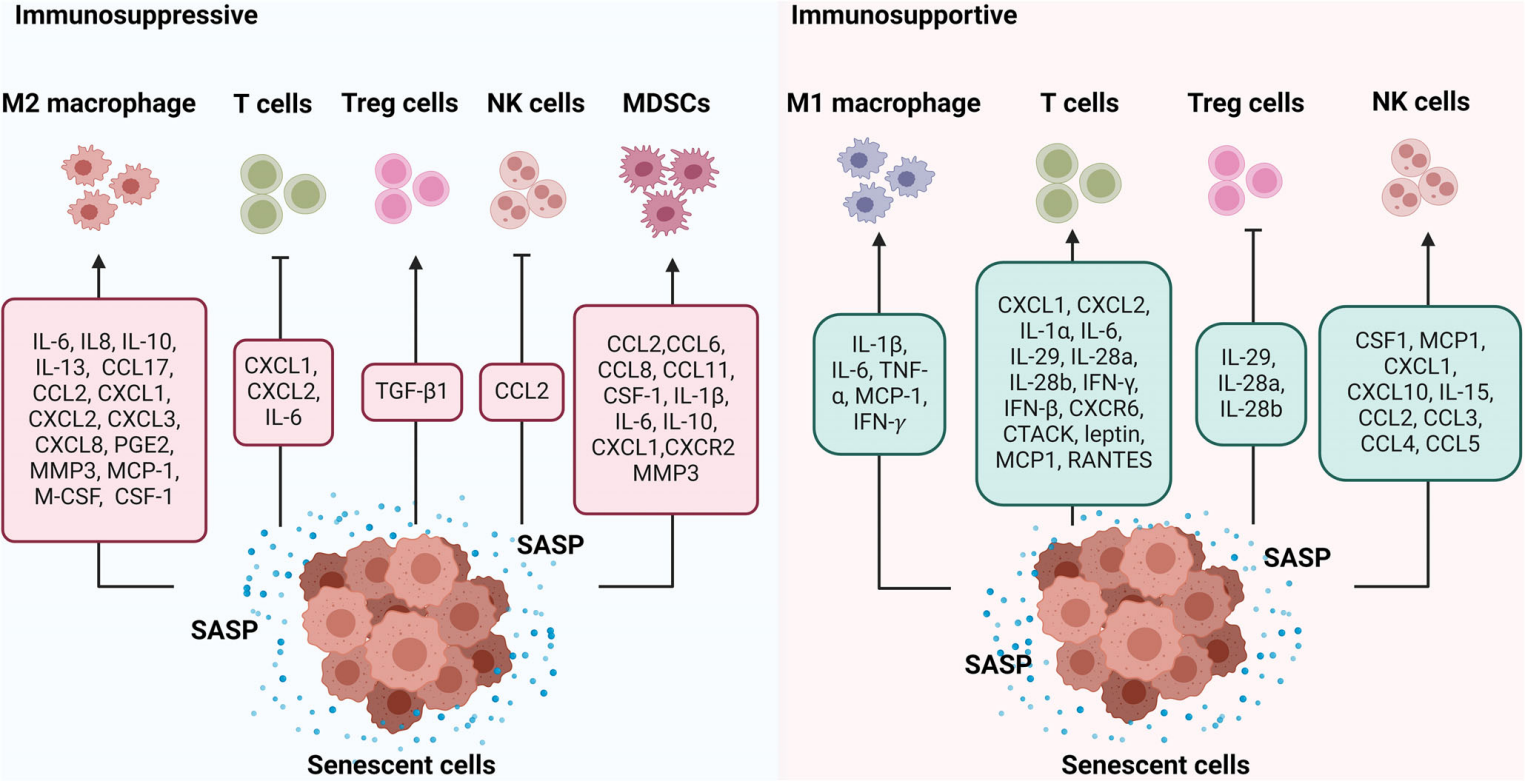
The SASP plays diverse roles in remodelling the tumour immune microenvironment. Certain SASP components like IL‐6, IL‐8, IL‐10, IL‐13, CCL17, CCL2, CXCL1, CXCL2, CXCL3, CXCL8, PGE2, MMP3, MCP‐1, M‐CSF and CSF‐1 promote the infiltration of M2 macrophages, while others like IL‐1β, IL‐6, TNF‐α, MCP‐1 and IFN‐γ also promote M1 macrophage infiltration. Additionally, CXCL1, CXCL2, IL‐1α, IL‐6, IL‐29, IL‐28a, IL‐28b, IFN‐γ, IFN‐β, CXCR6, CTACK, leptin, MCP1 and RANTES facilitate T cell infiltration, although CXCL1, CXCL2 and IL‐6 may inhibit T cell infiltration. TGF‐β1 induces the differentiation of Treg cells, while IL‐29, IL‐28a and IL‐28b inhibit Treg cell differentiation. Furthermore, CCL2 inhibits NK cell activity, whereas CSF1, MCP1, CXCL1, CXCL10, IL‐15, CCL2, CCL3, CCL4 and CCL5 promote NK cell activity. CCL2, CCL6, CCL8, CCL11, CSF‐1, IL‐1β, IL‐6, IL‐10, CXCL1, CXCR2 and MMP3 promote the infiltration of MDSCs.

In summary, the intricate interaction between senescent tumour cells and T cells, mediated by the SASP, has multifaceted effects on tumour progression (Figure 3). These findings underscore the potential for novel therapeutic strategies that leverage the immune‐modulating properties of SASP to enhance anti‐tumour immune responses.

#### 4.1.3 SASP and NK cells

NK cells, formidable warriors of innate immunity, play a pivotal role in the initial defence against cancer. Recent research has illuminated their central role in the surveillance of senescent cells, unveiling their crucial contribution to immune responses.

In hepatoma cell cultures, SASP factors, including CSF1, MCP1, CXCL1 and IL‐15, as well as adhesion molecules such as Icam1 and Vcam1, have been observed to attract a spectrum of immune cell types, encompassing macrophages, NK cells and neutrophils. This mobilisation of immune cells fosters natural immunity, contributing to the inhibition of tumour through direct cytotoxicity and phagocytosis.^100^ Furthermore, in the context of senescent liver cancer cells induced by doxycycline treatment, including CCL2, CCL3, CCL4 and CCL5, serves to recruit NK cells. These recruited NK cells effectively eliminate senescent tumour cells, thereby impeding tumour development.^16^ In a separate study involving RB‐mediated senescent lung cancer cells induced by MAPK and CDK4/6 inhibitors, increased expression of CCL2, CCL4, CCL5, CXCL10 and CXCL1 was observed. These chemokines played a critical role in attracting NK cells to the TME, where they facilitated the immune clearance of senescent tumour cells, thereby achieving effective tumour control.^48^ However, it should be noted that senescent hepatocytes can have a contrasting effect. They can inhibit NK cell activity through the secretion of CCL2, which, paradoxically, promotes the growth of established HCCs.^17^ This dual role underscores the complexity of interactions within the TME, where senescent cell‐secreted chemokines can have opposing effects depending on the stage of cancer development. These findings illuminate the complex dynamics between senescent cells and immune responses in the TME, offering insights into potential therapeutic strategies harnessing NK cell‐mediated immunosurveillance (Figure 3).

#### 4.1.4 SASP and MDSCs

MDSCs have emerged as significant contributors to tumour progression, playing pivotal roles in immune suppression, tumour angiogenesis, drug resistance and the facilitation of tumour metastasis.

The impact of MDSCs on tumours primarily stems from their effects on other immune cell, such as T cells, NK cells and B cells, resulting in immunosuppression and the promotion of tumour progression.^105^ In a murine model of HCC, CCL2 secreted by senescent cells, is implicated in the recruitment of MDSCs, which, in turn, inhibit NK cell function, creating a pro‐tumour environment.^17^ Similarly, in senescent prostate cancer cells induced by Pten gene loss, the SASP, encompassing factors such as IL‐1β, IL‐6, IL‐10, CXCL1 and CSF‐1, attracts MDSCs. These MDSCs, in their immunosuppressive role, hinder cytotoxic responses of NK and T cells while facilitating the transition to prostate adenocarcinoma.^18^ Notably, stromal senescence induced by p27 expression in fibroblasts can drive tumour progression through the IL‐6‐mediated recruitment of MDSCs, which subsequently impede anti‐tumour cytotoxic T cell responses.^40^ Moreover, normal fibroblasts treated with palbociclib, a CDK4/6 inhibitor, enter senescence and produce a SASP under the influence of NF‐κB. This SASP promotes the infiltration of MDSCs and enhances melanoma growth.^101^ Importantly, clinical development of CXCR2 inhibitors has demonstrated the capacity to restrain MDSCs infiltration following docetaxel‐induced senescence in prostate cancer models, offering a potential therapeutic avenue.^106^

In conclusion, MDSCs exert significant influence on tumour dynamics by modulating the immune responses of various immune cell subsets. SASP primarily functions to promote the inhibition of MDSCs, thereby facilitating tumour growth (Figure 3). Understanding these interactions may lead to novel therapeutic strategies aimed at disrupting the SASP‐mediated immune suppression within the TME.

### 4.2 SASP‐induced vascular remodelling

Cancer cells depend on oxygen and nutrients for their growth, which are provided by the vasculature, and angiogenesis is a vital step for the growth and dissemination of solid tumours.^107^ Senescent cells produce high levels of pro‐angiogenic SASP factors, including VEGF, PDGF and FGF, resulting in vascular remodelling in tumour (Figure 2).

Early studies demonstrated that senescent fibroblasts secrete VEGF, fostering angiogenesis and propelling breast cancer tumorigenesis and progression in immunocompromised mice.^108^, ^109^ In another study, senescent human peritoneal mesothelial cells were found to modulate the secretory profile of ovarian cancer cells, specifically augmenting the secretion of four key angiogenic factors: CXCL1, CXCL8, HGF and VEGF, resulting in an enhanced angiogenic potential of the vascular endothelium.^110^ In an animal model of pancreatic ductal adenocarcinoma, the combination therapy of trametinib and palbociclib induced tumour cell senescence and resulted in a significant secretion of SASP factors, including pro‐angiogenic factors (VEGF, PDGFA/B, FGF2), which in turn activated vascular endothelial cells and promoted vascular remodelling.^50^ Notably, a critical outcome of SASP induction is the up‐regulation of VCAM‐1, a cell surface protein that facilitates lymphocyte adhesion and extravasation into tissues. In HCC, SASP components, including IL‐6, IL‐8, CXCL10 and AREG, foster angiogenesis, while overexpression of DNASE1L3 inhibits tumour angiogenesis by disrupting the SASP in response to DNA damage stress.^111^ In colon cancer, adiponectin promotes stromal cell senescence, partly facilitating angiogenesis and tumour growth through the secretion of CXCL1.^112^

While SASP‐mediated blood formation may promote tumour growth, it can also stimulate the immune response by enhancing the infiltration of immune cells into tumours. SASP factors like IL6, IL8, CXCL1 and ICAM1, secreted by senescent HCC cells, induce the expression of NF‐κB in endothelial cells. This induction promotes lymphocyte recruitment, resulting in cytotoxic effects and the inhibition of tumour growth.^113^

Collectively, based on the aforementioned studies, the SASP can induce vascular remodelling, which may either facilitate tumour cell intravasation or, conversely, promote immune cell infiltration into tumours. Further research is warranted to fully elucidate the roles of pro‐angiogenic SASP and vascular remodelling in immune responses across various cancer contexts.

### 4.3 SASP‐induced ECM remodelling

The ECM plays a pivotal role in tumour progression by undergoing alterations in macromolecular components, degradation enzymes and stiffness. In the majority of tumour tissues, ECM remodelling is marked by heightened collagen synthesis and deposition, often coincident with the up‐regulation of various remodelling enzymes, including MMPs, lysyl oxidases (LOX and LOXLs) and proteins from the WNT1‐inducible signalling pathway. ECM remodelling exerts a profound impact on various aspects of tumour development, including proliferation, metastasis, angiogenesis and immune evasion.

Cancer‐associated fibroblasts (CAFs) are the principal orchestrators of ECM remodelling.^114^ The paracrine tumorigenic activity of senescent cells was first discovered when senescent fibroblasts were found to promote cancer cell proliferation.^108^ In murine ovarian cancer models, senescent fibroblasts induced by growth‐regulated oncogene 1 (Gro‐1) were shown to enhance tumour growth.^115^ In tumour xenografts, the expression of multiple characteristic SASP factors, including GATA4, MMP2/10, ITGA2 and GBP1, significantly increased after PRC2 inhibition, contributing to ECM remodelling and tumour regression.^116^ In human breast cancer cells, the expression and secretion of CXCL10, a newly identified SASP factor, promoted ECM invasion and tumour progression.^117^ In the context of oral malignant tumours, SASP originated from myofibroblasts triggers epithelial–mesenchymal transition (EMT) in oral submucous fibrosis and promotes tumour progression.^118^ The studies above indicate that the SASP derived from senescent cells can lead to ECM remodelling, thereby contributing to tumour progression (Figure 2).

This inflammatory state induced by SASP facilitates ECM deposition, which, in turn, induces contraction and increases stiffness.^119^ Elevated stiffness enhances tumour growth, EMT transition, invasion and metastasis through mechanical signal transduction pathways responding to biomechanical signals in the TME, thereby promoting breast cancer progression.^120^ In HCC, increased substrate stiffness promotes tumour proliferation and chemoresistance through a pathway involving β1‐integrin and focal adhesion kinase.^121^ The SASP from senescent CAFs induce alterations in recently‐deposited ECM proteins, thereby further increasing ECM stiffness, ultimately fostering metastasis and therapeutic resistance.^122^ Conversely, targeting collagen stabilisation reduces tumour stiffness, which can enhance T cell migration and improve the efficacy of anti‐PD‐1 therapy.^123^

Current research predominantly focuses on studying the impact of SASP factors secreted by senescent fibroblasts under the influence of the TME on tumour progression. The detailed mechanisms underlying the regulation of SASP factors on fibroblast function and ECM remodelling warrant further exploration.

### 4.4 SASP‐induced CSCs reprogramming

CSCs possess the unique ability to self‐renew and differentiate, and they play an essential role in promoting tumour growth, therapy resistance, distant metastasis and tumour recurrence. It is noteworthy that senescence and stemness are co‐regulated through interrelated signalling pathways, encompassing key factors such as p16, p21 and p53. This suggests that senescence can trigger the up‐regulation of stemness properties, thereby contributing to CSCs‐driven tumour progression and the development of therapy resistance.^124^

In a comparative gene expression analysis of senescent versus non‐senescent B‐cell lymphomas derived from Myc transgenic mice, a notable increase in the expression of specific stemness markers was detected in the senescent cells.^125^ Importantly, the onset of senescence in acute myeloid leukaemia and acute lymphoblastic leukaemia has been observed to convert non‐stem bulk leukaemia cells into self‐renewing leukaemia‐initiating stem cells. Additionally, administration of doxorubicin results in the induction of senescence in hepatoma cells, concomitant with a notable up‐regulation of stemness‐related gene, including SOX2, EpCAM, KLF4, CK19, c‐MYC and ANXA3.^126^

SASP has the potential to induce the reprogramming of cancer cells to escape from apoptosis, leading to the formation of CSCs. For example, IL‐6 and IL‐8, which are quintessential pro‐inflammatory cytokines within the SASP, are pivotal in sustaining CSCs (Figure 2). IL‐6 and IL‐8 treatment in MCF‐7 cells, either individually or in combination, induces fibroblastoid morphology, up‐regulates CD44 expression, enhances migration, self‐renewal and multilineage differentiation capacity, all of which are consistent with an EMT program and stemness.^127^ Notably, neutralising antibodies against IL‐6 and IL‐8 can reverse the effects of senescent cell‐conditioned medium on the breast cancer cell line MCF‐7, highlighting these cytokines act as pivotal mediators of EMT and stemness‐related effects within the senescent microenvironment. Furthermore, IL‐6 participates in the inducible formation of CSCs, regulating the CSC‐associated OCT4 gene expression via the IL‐6/JAK1/STAT3 signal axis.^128^ In another study, MCF‐7 cells, which initially exhibit a low stemness index, may acquire some stem‐like attributes after stimulation with IL‐8, ultimately increasing their aggressiveness.^129^ In colon CSCs, low concentrations of IL‐8 inhibitors and CXC receptor 2 (CXCR2) inhibitors effectively attenuate CSCs' stemness and facilitate the re‐entry into the cell cycle of dormant CSCs within the co‐culture system.^130^ In a recent study, SASP, such as ICAM1, AREG, MMP3, MMP10 and CTGF, is reported to promote tumorigenesis and progression of intrahepatic cholangiocarcinoma through enhancing the stemness of CSCs.^131^

Elucidating the molecular pathways that govern these reprogramming mechanisms during cellular senescence holds the potential to help overcome the challenges faced by contemporary therapies in effectively targeting CSCs in the future. Extensive studies across various cancer types are warranted to determine whether the reversal of senescence occurs is dependent on the specific cancer type, and it is essential to identify specific markers indicative of reversible senescence.

## 5 SENOTHERAPEUTICS FOR TUMOUR THERAPY

Incorporating senescence‐inducing therapies alongside other agents to selectively eliminate harmful senescent cells or adjust SASP factors in different cancer contexts may improve cancer therapy. Senolytic, a class of compounds or drugs, are specifically designed to induce apoptosis in senescent cells. Alternatively, even without directly eliminating senescence, the use of drugs to inhibit the secretion of SASP factors, referred to as senomorphic, holds promise for anti‐cancer therapy (Table 2). Furthermore, some targeted therapeutic approaches, including targeting senescent cells, nanotherapy and senolytic vaccines, are also utilised in anti‐tumour treatments.

**TABLE 2:** Senolytic and senomorphic therapy for cancer.

Drug	Molecular targets	Functional mechanism	Cancer type	Current status	References
Dasatinib	Pan‐receptor tyrosine kinases	Apoptosis	Haematological neoplasm, breast, gastric, prostate cancer	Preclinical animal models/clinical trials	^132^
Quercetin	PI3K/Akt	Apoptosis	Breast, prostate, lung, colorectal, pancreatic, melanoma cancer	Preclinical animal models/clinical trial	^133^
Fisetin	PI3K/Akt/mTOR	Apoptosis	Colon, gastric, prostate, pancreatic cancer	Preclinical animal models/clinical trial	^134^
ABT‐263	BCL‐2, BCL‐W, BCL‐xL	Apoptosis	Lung, leukaemia, oral, glioblastoma, eosophageal, breast, ovary cancer	Preclinical animal models/clinical trials	^135^, ^136^, ^137^, ^138^
ABT‐737	BCL‐2, BCL‐W, BCL‐xL	Apoptosis	Leukaemia, lung colon, glioblastoma cancer,	Preclinical animal models	^139^, ^140^, ^141^, ^142^, ^143^, ^144^
Ouabain	Na+/K+‐ATPase	Apoptosis	Liver, breast, colon, melanoma cancer	Cell‐based assays	^145^, ^146^
FOXO4 peptide	FOXO4‐p53	Apoptosis	p53‐wt cancer cells	Preclinical animal models	^147^
Procyanidin C1	AKT, JAK1/2	Apoptosis	Prostate cancer	Cell‐based assays	^148^
Rapamycin	mTOR	Inhibit SASP	Prostate, breast cancer	Cell‐based assays	^149^
Roxadustat	mTOR	Inhibit SASP	Breast cancer	Cell‐based assays	^150^
CDD111, ATI‐450	P38‐MK2	Inhibit SASP	Breast cancer	Cell‐based assays	^151^
NVP‐BSK805	JAK2/STAT3	Inhibit SASP	Prostate cancer	Preclinical animal models	^18^
Anakinra	IL‐1R	Reduce IL‐6 and IL‐8	Breast cancer	Cell‐based assays	^152^
Adalimumab	TNF‐α	Reduce TNF‐α	Breast cancer	Cell‐based assays	^153^
Siltuximab	IL‐6	Reduce IL‐6	Prostate, ovary, renal, colorectal cancer	Clinical trial	^154^
Tocilizumab	IL‐6R	Reduce IL‐6	Ovary, oral, lung cancer	Clinical trial	^154^

### 5.1 Senolytic therapy

Dasatinib and Quercetin (D+Q), two powerful senolytics in age‐related disease, could induce tumour cell apoptosis.^11^, ^155^ Fisetin, a natural flavonoid compound similar in structure and function to Quercetin, has been used in cancer therapy extensively.^134^ Procyanidin C1 (PCC1), a polyphenolic compound found in grapefruit seed extract, exhibits a dual capability by promoting apoptosis in senescent cells and inhibiting SASP expression in prostate cancer, leading to tumour regression and reduced chemoresistance.^148^

BCL‐2 inhibitors, including ABT‐263 (Navitoclax) and ABT‐737, can induce the apoptosis of senescent cells and have been extensively applied in the therapeutic management of a broad spectrum of cancers.^156^ ABT‐263 has demonstrated its ability to efficiently eliminate senescent ovarian, breast and lung cancer cells.^135^, ^136^, ^137^ In lung tumour‐bearing mice, ABT‐737‐induced clearance of senescent cells led to a markedly extended survival.^139^ In a mice model of pancreatic ductal adenocarcinoma, ABT‐737 treatment eliminated senescent cells dramatically reduced tumour development and progression.^140^

Ouabain, capable of binding to and impeding the transport activity of the Na+/K+‐ATPase, has been identified as a senolytic compound with the ability to eliminate senescent tumour cells.^145^, ^146^ Furthermore, forkhead box O4 (FOXO4) and p53 are recognised for their interplay in the apoptotic process of senescent cells. A specific molecule designed for this purpose is the FOXO4 peptide, which has demonstrated its ability to selectively eliminate senescent cells by promoting apoptosis.^147^

### 5.2 Senomorphic therapy

The mTOR pathway is a crucial regulator of the SASP, primarily through the activation of NF‐κB. The mTOR inhibitor (rapamycin) intervention has limited the growth‐promoting impact of senescent fibroblasts on prostate tumours.^157^ Rapamycin may also exert its control over the SASP by selectively modulating the translational activity of MK2 kinase via 4EBP1.^149^ Compounds that mimic hypoxia, such as roxadustat, have been shown to reduce SASP and enhance the resilience of chemotherapy‐treated and aged mice.^150^ Inhibitors (CD111, ATI‐450) of the p38 pathway or its downstream kinase MK2 have also demonstrated the ability to suppress the SASP, resulting in reduced breast cancer metastasis.^151^ In addition, JAK2 inhibitor (NVP‐BSK805) treatment reduced the SASP expression to enhance docetaxel anti‐tumour effects.^18^

Beyond targeting signalling pathways, senomorphic therapy can directly focus on specific SASP factors or their receptors. For example, anakinra, an antagonist of the IL‐1 receptor (IL‐1R), has notably reduced the invasiveness of metastatic cancer cells through the reduction of IL‐6 and IL‐8 secretion by senescent cells.^152^ Adalimumab, a monoclonal antibody targeting TNF‐α, has modulated various SASP‐associated markers, mitigating the tumour‐promoting effects of SASP.^153^ Monoclonal antibodies targeting IL‐6 (Siltuximab) and the IL‐6 receptor (IL‐6R) (Tocilizumab) have emerged as promising immunotherapeutic agents, utilisable as standalone treatments or in conjunction with traditional chemotherapy, for tumour therapy.^154^, ^158^ These senomorphic approaches offer promising avenues for targeted therapies that selectively modulate the SASP to improve cancer treatment outcomes.

### 5.3 Targeted therapies for tumour senescence

#### 5.3.1 Nanomedicine‐based senescence‐associated treatment

Nanomedicine offers substantial benefits in the realm of cancer treatment, including enhanced drug delivery, synergistic combination therapies, precise targeted therapy as well as the longer circulation time. Capitalising on these attributes, a nanomedicine‐based strategy has been employed to address senescence‐associated challenges and thus improve the efficacy of cancer treatment.

**FIGURE 4:**
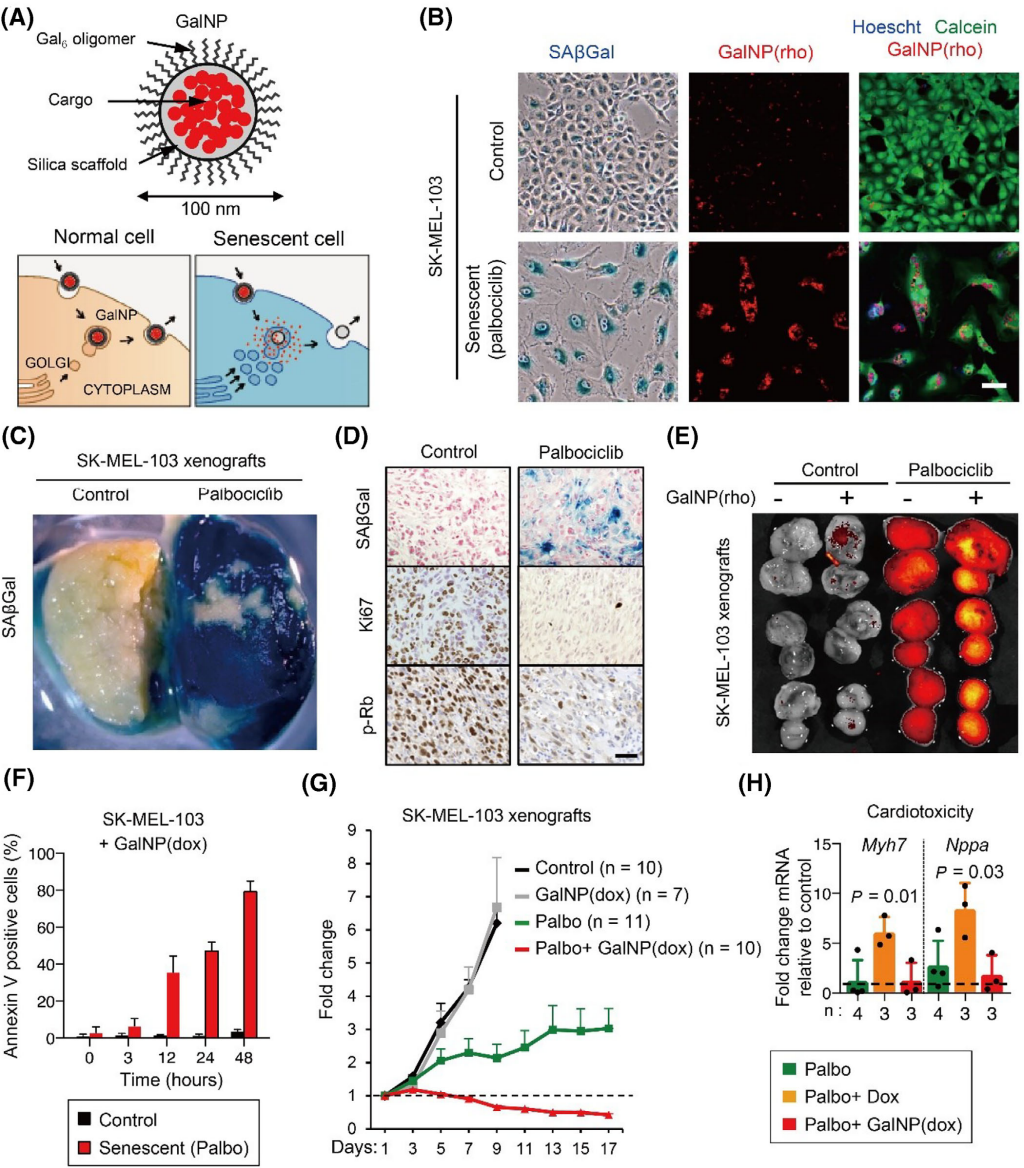
Gal‐encapsulated NPs for tumour therapy. (A) Schematic diagram of GalNPs nano‐drug design targeted specifically for senescent tumour cells. B, the release of fluorescent dyes in senescent SK‐MEL‐103 cells due to SA‐β‐gal‐mediated hydrolysis of rhodamine‐loaded GalNP. (C and D) SA‐β‐gal staining and immunohistochemistry staining of tumour tissue treated with palbociclib in SK‐MEL‐103 xenografts. (E) The fluorescence of GalNP(rhodamine) analysed using an IVIS spectrum imaging system. (F) Apoptosis detection in senescent SK‐MEL‐103 cells treated with GalNP(doxorubicin). (G) The relative tumour volume change in SK‐MEL‐103 xenografts treated with different schemes. (H) The relative mRNA changes of cardiotoxicity markers (Myh7 and Nppa) in the different groups (the figure is reproduced from Daniel et al. DOI: 10.15252/emmm.201809355).

**FIGURE 5:**
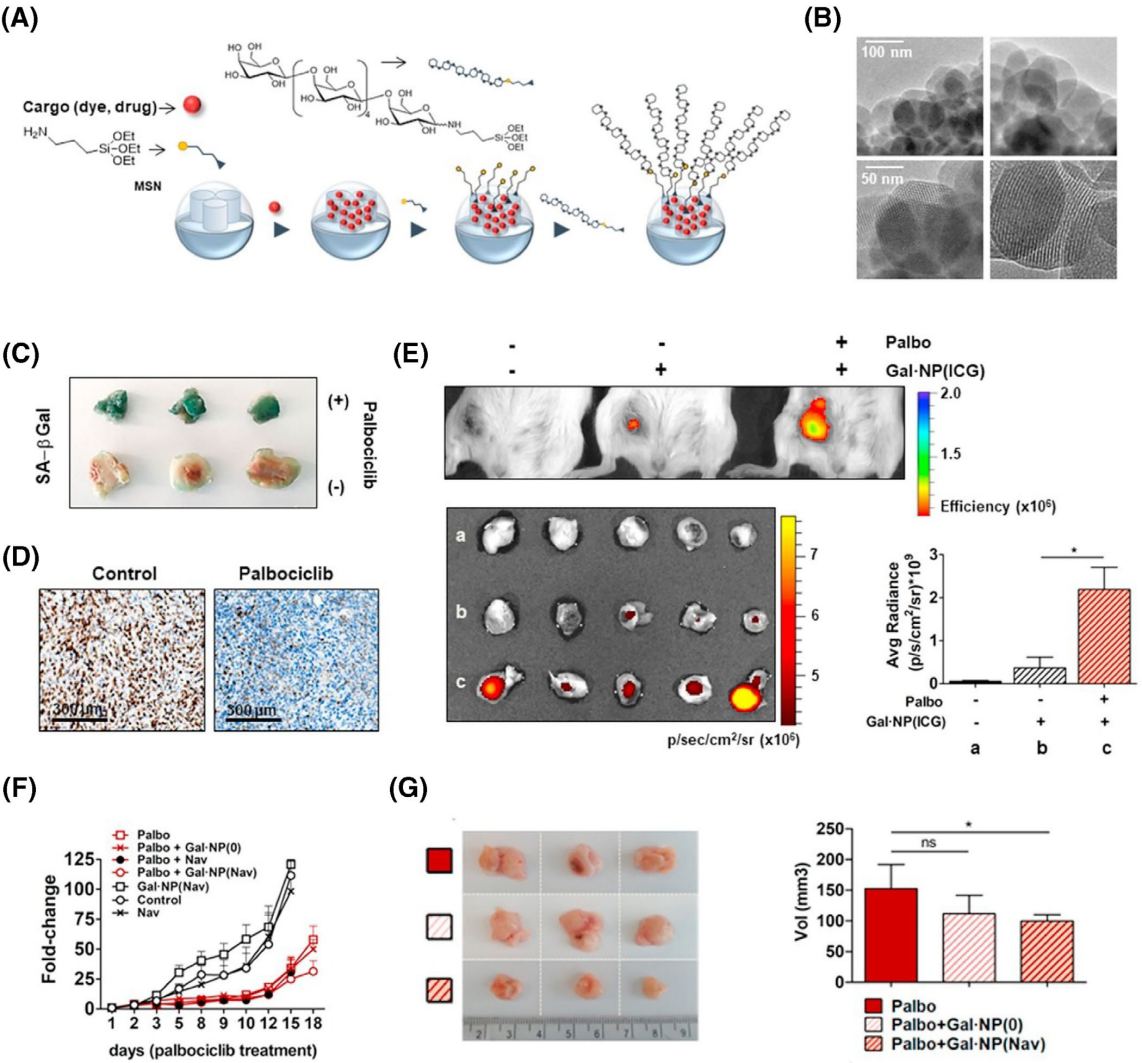
Gal‐encapsulated NPs for the treatment of breast cancer. (A) Schematic diagram of GalNPs nano‐drug design targeted specifically for senescent tumour cells. (B) Transmission electron microscope (TEM) images of GalNP. (C and D) SA‐βgal staining and immunohistochemistry staining (Ki‐67) of tumour tissue treated with palbociclib in 4T1 breast cancer xenografts. (E) The fluorescence of GalNP(ICG) analysed using an IVIS spectrum imaging system. (F) The relative tumour volume change in 4T1 xenografts treated with different schemes. (G and H) The analysis of tumour volumes for selected treatment approaches (the figure is reproduced from Irene et al. https://doi.org/10.1016/j.jconrel.2020.04.045).

A nano‐drug formulation utilising the third‐generation poly (amidoamine) (PAMAM) dendrimer G3 has been conjugated with lapatinib—a potent dual inhibitor of the EGFR and HER2 tyrosine kinases—and fulvestrant, a selective ER degrader. This innovative approach was designed to enhance apoptosis in breast cancer cells that have undergone senescence as a result of doxorubicin treatment.^159^ For the specific targeting of chemotherapy‐induced senescent tumour cells in SK‐MEL‐103 melanoma xenograft models, silica‐based nanoparticles encapsulating cytotoxic drugs conjugated to galacto‐oligosaccharides are engineered to release their payload upon interaction with the enzymatic activity of SA‐β‐gal within these senescent cells (Figure 4). This strategy, in tandem with palbociclib, a recognised CDK4/6 inhibitor with anti‐cancer effects, enhances the therapeutic approach to tumour treatment.^160^ Additional, to mitigate the systemic toxicity associated with senolytic navitoclax while enhancing the clearance of senescent breast cancer cells, nano‐encapsulated navitoclax conjugated to galacto‐oligosaccharides demonstrated significant anti‐tumour efficacy alongside a reduced side effect profile (Figure 5).^161^ Furthermore, to enhance the uptake efficiency of nano‐drugs by senescent cells, calcium carbonate nanoparticles conjugated with a CD9 monoclonal antibody was designed since CD9 surface receptors are up‐regulated in senescent cells, thereby potentially increasing the specificity and effectiveness of the nano‐drug delivery.^162^ In addition, similar to nanoparticles, EV‐based senolytic strategies can also be shown to eliminate senescent cells. For instance, engineered senolytic EVs, conjugated with anti‐GPNMB antibodies and loaded with the combination drug D+Q, have demonstrated efficient and selective eradication of senescent tumour cells, thereby activating anti‐tumour immunity in a murine model.^163^

Currently, a multitude of senescent cell characteristics—such as SA‐β‐gal activity, cell surface receptor expression, intracellular senescence‐associated signalling pathways, differentially expressed proteins and methylation patterns in DNA—have been identified and harnessed for the detection and treatment of senescent cells. These advancements are facilitated by a range of sophisticated techniques, including the use of nano‐drugs, fluorescent probes, positron emission tomography (PET) imaging agents and epigenetic analysis of modified DNA samples.^164^ Furthermore, a groundbreaking first‐in‐man study was conducted using a SA‐β‐gal specific PET probe in conjunction with alisertib treatment. The findings indicated that there was a high and heterogeneous uptake of the probe in regions of liver metastasis, suggesting the potential for PET imaging to effectively monitor and eliminate the senescent cells within tumours.

The nano‐based drug delivery system provides innovative therapeutic strategies for eliminating senescent cells. To validate the efficacy of these advanced senolytics against senescent cells, additional research is essential, along with their evaluation in both preclinical and clinical contexts.

#### 5.3.2 Immunotherapy to combat cellular senescence

uPAR serves as a cell‐surface protein that is markedly up‐regulated during cellular senescence across both neoplastic and non‐neoplastic tissues. Chimeric antigen receptor (CAR)‐T cell therapies, engineered to specifically recognise uPAR, hold promise as potent senolytic agents capable of eliminating senescent cells. CAR‐T cells engineered to specifically target uPAR have demonstrated the ability to significantly prolong the survival of mice with lung adenocarcinoma, particularly when cellular senescence is induced by treatment with MEK and CDK4/6 inhibitors.^165^ Furthermore, the NKG2DLs are consistently up‐regulated on senescent cells, irrespective of the stimuli that initiating senescence. This up‐regulation is also observed in vivo within the tissues of aged mice and nonhuman primates. Targeting senescent cells with NKG2D‐CAR‐T cells has been demonstrated to ameliorate a range of aging‐associated pathologies and enhance physical performance in both irradiated and aged mice, underscoring the therapeutic potential of these engineered immune cells in combating the deleterious effects of cellular senescence.^166^

GPNMB, identified as an additional potential target, is characterised as a senoantigen within senescent endothelial cells. An immunisation strategy using two peptide sequences from the extracellular domain of GPNMB conjugated with Titer Max Gold were selected to create senolytic vaccines against high fat diet‐induced atherogenesis in mouse models. The targeted clearance of senescent cells with elevated GPNMB expression showed not only alleviate both normal and pathological aging processes in aged mice but also to significantly extend the lifespan of mice exhibiting premature aging phenotypes.^167^

While immunotherapy presents a novel and promising approach for targeting senescent cells in cancer treatment, it faces challenges such as cost and side effects. Consequently, there is a necessity for further in‐depth research to develop more effective immunotherapeutic methods for the elimination of senescent cells.

## 6 DISCUSSION

Tumours and their microenvironments are intricately interconnected, with tumours releasing signalling molecules that influence the TME. Traditional cancer therapies, including chemotherapy and radiotherapy, can induce senescence in cancer cells accompanied by the secretion of SASP factors, which has long been considered a key mechanism for suppressing tumour growth. However, recent research has unveiled that senescent cancer cells and SASP can have direct or indirect effects on remodelling the TME, ultimately promoting tumour recurrence and metastasis. Cancer therapy may also induce senescence in non‐malignant cells, particularly immune cells, which can result in weakened immune surveillance and promote tumour growth. Moreover, non‐malignant cells with lasting senescence can assume characteristics that actively contribute to the tumorigenic process.

Accumulation of cellular damage in tumour cells undergoing senescence results in the SASP, which in turn affects both the tumour itself and its surrounding environment. The TME accumulates cytokines that can disturb the gene expression of senescent tumour cells, potentially leading to abnormal gene expression and dynamic changes in SASP composition. This rearranged SASP can have diverse effects on immune cells and the tumour process. Furthermore, SASP‐induced vascular remodelling can either facilitate tumour cell intravasation through blood vessels or promote immune cell infiltration into tumours. Additionally, SASP‐induced ECM remodelling can drive EMT and support cancer progression. Senescence‐associated stemness activation can contribute to CSCs‐induced tumour relapse and metastasis.

The current evidence suggests that specifically targeting senescent cells or reducing SASP production may offer a therapeutic opportunity to complement traditional cancer therapy approaches, limiting the incidence and progression of cancer. Senolytic therapy, which drives senescent tumour cells into apoptosis, can be deployed in conjunction with senescence‐inducing agents. However, traditional senolytic agents often encounter issues related to their multiple‐target and off‐target effects. For instance, dasatinib, a multi‐target tyrosine kinase inhibitor, exhibits a broad spectrum of activities that may lead to systemic toxicity. Similarly, navitoclax has been linked to significant haematological toxicities, affecting neutrophil and platelet counts. Quercetin presents with low bioavailability, which limits its therapeutic potential. Consequently, safety considerations are of utmost importance, especially when medications are given systemically. To address these concerns and minimise the toxic side effects associated with senotherapy, intermittent administration regimen has been proposed. It may significantly reduce the off‐target effects, thereby enhancing the safety profile of senolytic treatments. Additionally, localised injection therapies have the potential to provide effective treatment while minimising systemic adverse reactions. For example, topical application, particularly for senolytics with low oral bioavailability, could represent a promising approach for managing senescence‐related skin conditions, such as melanotic nevi or psoriasis.^168^ The search for natural products with potent therapeutic effects and minimal side effects, like PCC1, represents a significant area of research. Further studies are essential to confirm its potential within the field of tumour senotherapy.

In recent years, remarkable advancements have been achieved in the field of targeted senotherapy, capitalising on the unique characteristics of senescent cells. Biomarkers such as SA‐β‐gal and cell surface markers including CD9, CD36, CD47, uPAR and B2M have been instrumental in this endeavour. The application potential of therapies that exploit these senescence‐associated features is considerable. As a result, a variety of targeted therapeutics, including small‐molecule prodrugs that target SA‐β‐gal, nano‐drugs, personalised CAR‐T therapies and senolytic vaccines, have been extensively utilised in preclinical studies. These approaches have demonstrated remarkable precision in targeting senescent cells. Despite considerable advances, the challenge remains that no single biomarker has been found to be unequivocally for senescent cells. The specificity of these markers can fluctuate according to cell type, tissue, species, disease stage and other variables. Consequently, a multi‐labelling strategy that employs two or more biomarkers, in conjunction with proliferation markers like Ki‐67 or BrdU, is advised to facilitate more precise in situ identification of senescent cells. Currently, senotherapy for tumour treatment is still in its infancy, translating these promising treatments to clinical practice faces challenges related to tumour type selection, safety and effectiveness.

In conclusion, the role and consequences of cell senescence in tumours are complex. The carcinogenic and anti‐cancer effects of senescent tumour cells are regulated by interactions between a variety of SASP factors and the immune microenvironment. The influence of SASP is substantially dependent on the environment and cell type, resident immune cells and is variable at different stages of tumour development. For example, during the later stages of tumorigenesis, SASP‐induced immunosuppression fosters tumour growth, whereas in the early stages, it functions as a tumour‐suppressive mechanism. Furthermore, senescent tumour cells can lie dormant for extended periods, circumventing therapeutic interventions and presenting a risk for tumour resurgence. In this review, we have highlighted the significant impact of SASP on the TME and underscored the importance of developing therapeutic strategies that target senescent cells and SASP to combat cancer. Looking ahead, clarifying the exact role of senescent cells in tumour prognosis will remain the focus of research, particularly within the context of cancer patients.

## AUTHOR CONTRIBUTIONS

Meng Wu, Wei Zhang and Shixuan Wang participated in the conception and design of the review article. Jiaqiang Xiong, Lu Dong and Qiongying Lv conceived the manuscript, participated in writing and prepared the tables and figures. Meng Wu, Wei Zhang, Shixuan Wang, Yutong Yin, Jiahui Zhao and Youning Ke critically reviewed each version of the manuscript. All the authors confirmed the final version.

## CONFLICT OF INTEREST STATEMENT

The authors declare no conflict of interest.

## ETHICS STATEMENT AND CONSENT TO PARTICIPATE

Not applicable.

## Data Availability

Not applicable.
